# Cas9 modulates Campylobacter jejuni virulence traits inside intestinal epithelial cells

**DOI:** 10.1099/mic.0.001638

**Published:** 2025-12-05

**Authors:** Chinmoy Saha, Dior Beerens, Peter van Baarlen, Rogier Louwen

**Affiliations:** 1 Department of Medical Microbiology and Infectious Diseases, Erasmus MC University Medical Center Rotterdam, Rotterdam, Netherlands; 2 Host–Microbe Interactomics Group, Wageningen University, Wageningen, Netherlands

**Keywords:** Caco-2, *Campylobacter jejuni*, Cas9, cell envelope, ciprofloxacin, survival, TLR-2

## Abstract

The CRISPR-associated protein 9 (Cas9) produced by disease-associated strains of *Campylobacter jejuni* contributes to full virulence, including immune evasion and bacterial survival inside eukaryotic cells. In this work, we explored the role of *C. jejuni* Cas9 (CjeCas9) in cell envelope integrity, antibiotic resistance, intracellular survival inside Caco-2 intestinal epithelial cells and Toll-like receptor 2 (TLR-2) activation. We show that CjeCas9 modulates the permeability of the *C. jejuni* cell envelope, sialylated lipooligosaccharide expression and susceptibility to ciprofloxacin, the most commonly prescribed antibiotic to treat *C. jejuni* infections. Moreover, we reveal that WT production of CjeCas9 increased intracellular survival of *C. jejuni* inside Caco-2 intestinal epithelial cells by a factor of 550 compared to the respective *cas9* gene deletion mutant and that intracellular survival was associated with the activation of TLR-2. In conclusion, we established that CjeCas9 modulates *C. jejuni* (intracellular) virulence traits, including intracellular survival.

## Data Availability

The generated genetically modified organisms and plasmids can be provided by the Erasmus MC pending scientific review and a completed material transfer agreement. Requests for genetically modified organisms and plasmids should be submitted to the Department of MMIZ, Erasmus MC. All data needed to evaluate the conclusions in the paper are present in the paper and/or the Supplementary Materials. Additional Supplementary Material is available with the online version of this article, available through Figshare at 10.6084/m9.figshare.30788381 [1] Additional data related to this article may be requested from the authors.

## Introduction

Clustered regularly interspaced short palindromic repeats and CRISPR-associated genes (CRISPR-Cas) loci [2] encode a sequence-specific heritable adaptive immune system that employs nuclease activity and targets invading nucleic acids, such as bacteriophages and plasmids [3]. In addition to their function in bacterial immunity against ‘foreign’ (or non-self) genetic elements, CRISPR-Cas systems can be involved in transcription regulation [4], cell envelope integrity [5], innate immune evasion [6], host colonization [7], antibiotic resistance [8,9], inflammasome evasion [5] and cytotoxicity [10]. Currently, CRISPR-Cas systems are classified into two main classes, based on the configuration of their effector protein complexes [11]. Class 1 CRISPR-Cas systems assemble multiple Cas proteins and a CRISPR RNA (crRNA) to form an effector complex, whereas class 2 CRISPR-Cas systems utilize a single multi-domain Cas protein, CRISPR-associated protein 9 (Cas9), bound to crRNAs to target invading DNA [11]. Of note, the occurrence of class 2 CRISPR-Cas systems with their characteristic Cas9 protein is overrepresented in pathogenic bacteria, including medically relevant zoonotic bacteria such as *Campylobacter jejuni* [12]. To date, CRISPR-Cas systems have been found in almost 50% of bacterial and 90% of archaeal genome sequences [13].

*C. jejuni* are Gram-negative intestinal pathogens that infect human host cells and constitute a worldwide leading cause of bacterial gastroenteritis [14]. Symptoms of *C. jejuni*-associated gastroenteritis range from mild diarrhoea with(out) fevers to severe bloody diarrhoea [14]. Controlled human clinical challenge studies enrolling healthy human volunteers who consumed two virulent *C. jejuni* strains showed that human *C. jejuni* infections are associated with tissue inflammation and that time to fever onset is ~68 h post-infection (hpi) [15]. *C. jejuni* presence in human jejunal fluid contents could be demonstrated from 24 h after challenge, and stool cultures were positive for viable *C. jejuni* bacteria 48 to 72 h post-challenge and stayed positive for 1 week; illness symptoms usually appeared within 1 day [15]. *In vitro*, virulent *C. jejuni* strains invade human epithelial cells as soon as 15–20 min after challenge [16,17] and damage/kill Caco-2 intestinal epithelial cells 72 hpi [10]. Once intracellular, highly virulent *C. jejuni* evade the inflammasome and transcytose across the intestinal epithelial barrier [16,17]. These virulence traits are associated with the production of sialylated lipooligosaccharide (LOS) on *C. jejuni* cell envelope, by which *C. jejuni* isolates can be divided into two groups based on the absence or presence of sialylated LOS [17]. Isolates included in the latter group are highly pathogenic, cause more severe colitis and are associated with post-infectious complications [18,20]. Sialylated LOS was also found to be an important trait mediating *C. jejuni* antibiotic resistance, by affecting cell envelope permeability, via unknown mechanism(s) [21,22]. Cell envelope composition and permeability is of major biological relevance to host-associated bacteria since the envelope provides essential protection against environmental molecules including antibiotics [23]. We discovered that *C. jejuni* with sialylated LOS presented high resistance to phage infections, a phenotype associated with functional *C. jejuni* Cas9 protein (CjeCas9) and reduced size (or absence) of CRISPR arrays [24]. In more virulent *C. jejuni* isolates, inactivation of CjeCas9 strongly affected (i) transcytosis across intestinal epithelial cells, (ii) changes in sialylated LOS recognition by human serum-related antibodies and (iii) cytotoxicity [24]. We found that a CjeCas9-dependent, but guide RNA-independent virulence mechanism enabled highly virulent *C. jejuni* isolates to damage and kill eukaryotic cells, a phenotype that was cell type- and cell line-dependent [10]. Based on these outcomes from our studies set up to unravel the non-canonical functions of CjeCas9, we wondered whether some as yet unknown CjeCas9 activity affected intracellular survival of *C. jejuni* in intestinal epithelial cells. This conceptual idea was based on previous findings observed in bacteria such as *Streptococcus agalactiae* and *Francisella novicida*, where the absence of Cas9 affected bacterial intracellular survival inside eukaryotic cells [4,5, 25]. We therefore explored, using a virulent *C. jejuni* isolate, the involvement of CjeCas9 in the modulation of cell envelope integrity, inflammasome evasion, intracellular survival and antibiotic resistance of *C. jejuni*.

## Experimental procedures

### Bacterial strains

We used the well-characterized *C. jejuni* WT strains GB11 and NCTC11168 (both encoding Cas9), their isogenic *cas9* gene deletion (Δ*cas9*) mutants and their corresponding Δ*cas9*::cas9-complemented mutant (encoding Cas9), respectively. To further assay the effect of *cas9* gene presence/absence on microscopic shape of *C. jejuni* bacteria during their interactions with intestinal epithelial Caco-2 cells, we used the *C. jejuni* WT strain 81-176 that lacks a CRISPR-Cas system including Cas9 and its GB11 *cas9* gene-supplemented variant, described in our previous work [24]. With respect to microscopic bacterial shape, under the standard culture conditions described below, two *C. jejuni* bacterial shapes can be easily identified using standard light or fluorescence microscopy (see below): the standard spiral (or helical) shape and a rod-like or coccoid shape. Extracellular coccoid *C. jejuni* show motility defects, have low infectious potential and display low adherence to epithelial cells in infection assays [26]. Details on generation and specific characteristics of these WT, mutant, complemented mutant and supplemented strains can be found in [24,27]. GB11 and its respective variants were recovered from original glycerol stocks and maintained by culturing on blood agar (BA) culture media containing 5% sheep blood (Becton Dickinson, Breda, Netherlands) and vancomycin (10 µg ml^−1^) (Sigma-Aldrich, Zwijndrecht, Netherlands). GB11Δ*cas9* was grown on BA media supplemented with vancomycin (10 µg ml^−1^) (Sigma-Aldrich) and chloramphenicol (20 µg ml^−1^) (Sigma-Aldrich). GB11Δ*cas9*::cas9 and 81-176 supplemented with the GB11 *cas9* gene were grown on BA media supplemented with vancomycin (10 µg ml^−1^) (Sigma-Aldrich) and erythromycin (10 µg ml^−1^) (Sigma-Aldrich); the NCTC11168Δ*cas9*::cas9-complemented mutant used in erythromycin and clarithromycin antibiotic sensitivity assays was grown on BA media supplemented with vancomycin (10 µg ml^−1^) (Sigma-Aldrich) and kanamycin (20 µg ml^−1^) (Sigma-Aldrich). All isolates were cultured under micro-aerophilic conditions at 37 °C, using anaerobic jars and an Anoxomat gas mixer (Mart Microbiology B.V., Drachten, Netherlands). To equilibrate growth conditions, 24 h before experimental use, strains were cultured on BA media supplemented with vancomycin (10 µg ml^−1^) (Sigma-Aldrich) unless stated otherwise.

### Eukaryotic cell cultures

The human epithelial colorectal adenocarcinoma cell line Caco-2 was maintained in Dulbecco’s Modified Eagle’s Medium (DMEM) (Invitrogen, Breda, Netherlands) supplemented with 10% FBS (Invitrogen), 100 U ml^−1^ penicillin, 100 U ml^−1^ streptomycin and 1% non-essential amino acid (NEAA) (Invitrogen). The HEK-Blue™ cell line, which is a human embryonic kidney (HEK)293 cell line transfected with human Toll-like receptor-2 (TLR-2), was maintained in DMEM (Invitrogen), supplemented with 10% FBS (Invitrogen), 1:100 dilution of a stock solution of penicillin-streptomycin (10,000 U ml^−1^), 100 mg ml^−1^ Normocin™ (InvivoGen, Toulouse, France), 2 mM l-glutamine and 1% NEAA (Invitrogen). Caco-2 and HEK cells were cultured and maintained in 25 or 75 cm^2^ flasks (Greiner Bio-One, Alphen a/d Rijn, Netherlands), respectively, at 37 °C and 5% CO_2_ in a humidified air incubator.

### Cell envelope permeability assays

Ethidium bromide and propidium iodide experiments were performed to check cell envelope permeability in the presence or absence of Cas9 as reported for *F. novicida* [5]. *C. jejuni* WT strains GB11, NCTC11168 (both encoding Cas9), their isogenic *cas9* (Δ*cas9*) gene deletion mutants and their corresponding Δ*cas9*::cas9-complemented mutants (encoding Cas9) were cultured as described above. Bacterial cells were harvested in Hank’s Balanced Salt Solution (HBSS) (Life Technologies, Zoetermeer, Netherlands) and washed in HBSS, upon which OD at 600 nm (OD_600_) was calibrated at 0.3, line. One hundred microlitres of bacterial suspension was then mixed with 100 µl of HBSS containing 30 µg ml^−1^ ethidium bromide or 100 µl HBSS containing 200 µM propidium iodide. Fluorescence intensity was measured immediately in a FluoStar Optima (BMG Labtech, De Meern, Netherlands) fluorescence meter at 605 nm for ethidium bromide or at 617 nm for propidium iodide.

### Determination of standard growth curves

To investigate the growth performance of WT and mutant strains, we carried out growth assays where GB11, NCTC11168 and their isogenic Δ*cas9* mutants were cultured in lysogeny broth (LB). In brief, WT and *cas9* mutants were grown overnight at micro-aerophilic conditions at 37 °C. After overnight growth, *C. jejuni* suspensions with OD (OD600_nm_) of 0.1 were prepared in LB, after which growth under micro-aerophilic culturing conditions at 37 °C was measured at OD600_nm_ for 52 h. Micro-aerophilic conditions were maintained by using anaerobic jars and an Anoxomat gas mixer (Mart Microbiology B.V.) plus a mixing plateau on which the jars were stably positioned in the incubator and gently shaken during incubation. We compared the growth of these strains to the growth performance of *C. jejuni* WT and isogenic *cas9* gene deletion mutant strains from our previous work [24].

### Antibiotic susceptibility assays

Susceptibility to commonly used bactericidal antibiotics, selected for their effect on bacterial cell envelope permeability, was tested using E-tests, ready-to-use reagent strips featuring 15 on-scale MIC (BioMérieux, Zaltbommel, Netherlands). *C. jejuni* WT strains GB11, NCTC11168, their isogenic *cas9* gene deletion (Δ*cas9*) mutants and corresponding Δ*cas9*::cas9-complemented mutants were cultured as described above and harvested in 0.9% saline solution (Sigma-Aldrich). *C. jejuni* suspensions were set at 0.5 OD_600_ in McFarland medium and swabbed onto Mueller-Hinton (MH) II agar media (Becton Dickinson). E-test strips (see above) for ciprofloxacin, tetracycline, colistin, polymyxin B, erythromycin and clarithromycin were positioned in the centre of solid agar culture media in petridishes and incubated overnight at 37 °C under microaerophilic conditions (see above). MIC values were determined after 24 h.

### Swarming behaviour assays

*C*. *jejuni* WT strains GB11, GB11 isogenic *cas9* (Δ*cas9*) mutant and corresponding GB11Δ*cas9*::cas9-complemented mutant were cultured as described above, harvested in HBSS (Life Technologies) and equalized at an OD600_nm_ of 1, corresponding with 2.5*10^9^ c.f.u. per ml. Semi-solid agar media (MH broth supplemented with 0.5% agar) were inoculated with 1 µl of bacterial suspension by stabbing [24]; inoculated plates were grown for 12–24h at 37 °C under micro-aerophilic conditions. Diameters of bacterial swarming were measured in millimetres. Detailed methods and previous results of *C. jejuni* swarming behaviour are in [24].

### Fluorescence microscopy

For recording motility and swimming patterns of *C. jejuni* WT and isogenic *cas9* mutant strains in liquid media, an XI51 phase-contrast fluorescence microscope (Olympus, Leiden, The Netherlands) was used. *C. jejuni* bacteria were diluted to OD600_nm_ of 0.2–0.4 in 2 ml pre-heated cell culture medium at 37 °C, and bacterial movements were recorded immediately after dilution. Movies were taken with an Olympus XM10 colour camera at ×40 magnification, saved as audio-video interleaved (avi) files and analysed using CellFˆ software (Olympus).

### Cholera toxin binding to sialylated LOS assays

*C. jejuni* WT strains GB11, GB11 isogenic *cas9* gene deletion (Δ*cas9*) mutant and corresponding GB11Δ*cas9*::cas9-complemented mutant were cultured as described above and harvested, washed and heat-inactivated for 45 min at 65 °C in HBSS (Life Technologies) supplemented with 2 mM MgCl2. Heat-inactivated bacteria were washed. Forty microlitres of this suspension containing 10^8^ heat-inactivated bacteria were incubated with 1 μg ml^−1^ FITC-labelled cholera toxin (CT) for 45 min at room temperature while shaking. FITC-labelled bacteria were washed twice with PBS, and mean fluorescence intensity (MFI) was measured with a FACS BD Accuri™ C6 flow cytometer (BD Biosciences, Vianen, Netherlands). FACS output data were analysed with BD Accuri C6 Software to test for significant fluorescence intensity differences between the strains analysed.

### Epithelial cell invasion assays

Invasion of epithelial cells by *C. jejuni* bacteria was assayed by growing monolayers of Caco-2 cells to 100% confluence in DMEM complete medium (10% FCS and 1× NEAA), without antibiotics in 12-well plates (Greiner Bio-One). Confluent Caco-2 monolayers were incubated at an m.o.i. of 100 with *C. jejuni* GB11, its isogenic Δ*cas9* gene deletion mutant and Δ*cas9*::cas9-complemented mutant strain for 4 h at 37 °C. After incubation, monolayers were washed three times in the wells by pipetting and removing pre-warmed HBSS. To kill extracellular bacteria, washed monolayers were treated for 2–3 h with a bactericidal dose of gentamycin (480 µg ml^−1^) (Sigma-Aldrich) in DMEM [28] in 10% FBS and 1× NEAA (Invitrogen). Antibiotic-incubated monolayers were washed 3× times with pre-warmed HBSS and Caco-2 cells were lysed with 0.2% Triton X-100 (Cornell, Philadelphia, PA) in pre-warmed HBSS for a max. of 15 min at room temperature. Of note, Triton X-100 does not influence intracellular survival of *C. jejuni* during this time span [17,28]. Serial dilutions of lysed cell solutions were prepared and plated onto pre-warmed BA media containing vancomycin (10 µg ml^−1^) (Sigma-Aldrich), and *C. jejuni* colonies were counted after 24–48 h of incubation and inspected for contamination. In case contamination was suspected, a Gram staining was prepared and checked at ×100 magnification using an Olympus microscope 84-000-0048 (Olympus, Hoofddorp, Netherlands). More details about the invasion protocol can be found in [28].

### *C. jejuni* survival assays

Caco-2 cells were cultured in DMEM medium without antibiotics in six-well plates until full confluency. Confluent Caco-2 cell monolayers were washed 3× with pre-warmed HBSS (37 °C) (Life Technologies) and incubated in pre-warmed DMEM medium containing 10% FBS, 1× NEAA without antibiotics (Invitrogen) and *C. jejuni* bacteria at m.o.i. of 100 for 4 h at 37 °C and 5% CO_2_ as described above. After incubation, Caco-2 cells were washed with pre-warmed HBSS (37 °C) (Life Technologies) and incubated for another 2 h in DMEM medium containing 10% FBS, 1× NEAA (Invitrogen) and gentamicin (480 µg ml^−1^) to kill extracellular bacteria. After gentamicin treatment, Caco-2 cell monolayers were washed twice with pre-warmed HBSS (37 °C), after which fresh DMEM medium containing 10% FBS, 1× NEAA and gentamicin (10 µg ml^−1^) was added. After 24 h incubation, Caco-2 cells were washed three times with HBSS pre-warmed at 37 °C and lysed in pre-warmed HBSS containing 0.1% Triton X-100 (total volume of 1 ml per well) for max. of 5 min at room temperature. Lysed Caco-2 cells were detached by gently up and down pipetting of 1 ml HBSS/0.1% Triton X-100 solution. Tenfold serial dilutions of the lysates were plated on BA agar media containing 5% sheep blood with vancomycin (10 µg ml^−1^) and incubated in anaerobic jars under microaerobic conditions using Anoxomat gas mixer (Mart) for 48 h at 37 °C, upon which numbers of CFUs were counted.

### Immunofluorescence and immunohistochemistry

Caco-2 cells were grown to 40%–50% confluency on chamber slides (Greiner Bio-One), whereafter *C. jejuni* GB11, its Δ*cas9* gene deletion mutant, WT strain 81-176 and 81-176 supplemented with the GB11 *cas9* gene were inoculated at an m.o.i. of 100. The latter two strains, 81-176 and its supplemented *cas9* variant, were used in this work to visualize bacterial cellular shape changes (see above, Bacterial strains) inside the eukaryotic cell in the presence or absence of CjeCas9 during staining with LAMP-1. We chose WT and GB11 Cas9-supplemented variant of strain 81-176 since we had previously shown that this supplementation improved intracellular survival of strain 81-176 by nearly a factor of 10 [17]. Our previous time course experiments revealed optimal timepoints for analysis of colocalization of the endosomal fluorescently labelled primary antibody markers EEA-1 (Biosciences BD, Breda, The Netherlands), RAB7 (Abcam, Cambridge, MA) and LAMP-1 (Abcam); these cellular markers are associated with invasion and survival of intracellular *C. jejuni* cells [17]. For EEA-1, incubation time after inoculation of *C. jejuni* into the chambers of the chamber slides was 15–30 min; for RAB7, 45 min; and for LAMP-1, 2 h, which were precisely determined earlier [17]. Prior to microscopy, Caco-2 cells were washed three times with pre-warmed (37 °C) HBSS (Invitrogen) and fixed with 4% formaldehyde (Sigma-Aldrich) in HBSS (Invitrogen) at room temperature for 2 h, whereupon Caco-2 cells were again washed three times with HBSS (Invitrogen). After the washing steps, Caco-2 cells were permeabilized for 20 min with 0.1% HBSS–Triton X-100 solution, and background antibody binding was suppressed with blocking buffer (1% FBS, 1% Tween-20, HBSS). Slides were incubated for 1 h with the respective primary antibody detecting EEA-1, RAB7 or LAMP-1 at a 1:100 dilution in block buffer. The appropriate secondary antibodies from the IgG class (H+L), A594 labelled (Molecular Probes, Bleiswijk, The Netherlands), providing a red stain, were selected for EEA1, RAB7 and LAMP-1 [17]. To visualize *C. jejuni*, an anti-*C. jejuni* FITC-labelled antibody (Genway, San Diego, CA) was diluted 1:100 in blocking buffer and incubated for 1 h [17]. Caco-2 cells were washed three times with HBSS (Invitrogen) and dehydrated for 1 min with 70% ethanol and another minute with 100% ethanol (Sigma-Aldrich, Zwijndrecht, The Netherlands). The culture chamber was removed from the slides, and the slides were air-dried. Slides with Caco-2 cells were mounted with fluorescent mounting medium (Dako, Carpinteria, CA), a coverslip was placed on top, and slides were analysed using an Olympus IX51 phase-contrast fluorescence microscope (Olympus). Images were captured with an Olympus XM10 colour camera at ×1,000 magnification, and Olympus software Cell-F was used to detect and count (i) the number of *C. jejuni* bacteria per cell, (ii) if bacteria were coccoid (see above) shaped and (iii) if bacteria colocalized with the respective endosomal markers upon invasion into Caco-2 cells. Experiments were independently repeated three times. In order to obtain the mean percentage of Caco-2 cell-associated *C. jejuni* bacteria per microscopic field, the number of intracellular coccoid-shaped and *C. jejuni* bacteria that colocalized with an endosomal marker was divided by the number of intracellular *C. jejuni* bacteria that did not colocalize with an endosomal marker, and the result was multiplied by 100.

### TLR-2 signalling assays

*C. jejuni* WT strain GB11, its *cas9* (Δ*cas9*) gene deletion mutant and corresponding GB11Δ*cas9*::cas9-complemented mutant were cultured overnight on BA media containing 5% sheep blood supplemented with vancomycin (10 μg ml^−1^) and harvested in HBSS at room temperature and calibrated at OD_600_=1, corresponding with 2.5*10^9^ c.f.u. per ml. After washing, bacteria were resuspended in DMEM without antibiotics and tenfold serial dilutions were prepared. Fifty microlitres of each dilution was added to each well of a 96-well flat-bottom tissue culture plate (Greiner Bio-One). Prior to signalling assays, hTLR-2 HEK-Blue™ cells were grown to 80% confluency in 11 ml DMEM without antibiotics, after which the hTLR-2 HEK-Blue™ cells were harvested and cell suspension aliquots of 200 µl were distributed across the 96 wells in DMEM medium without antibiotics. Wells containing semi-confluent hTLR-2 HEK-Blue™ cells were incubated with each of the respective prepared serial dilutions of one of the *C. jejuni* strains for 18 h at 37 °C. To assess TLR-2 signalling events, for each well, 20 µl of supernatant was mixed with 180 µl of pre-heated at 37 °C QUANTI-Blue™ substrate (InvivoGen) and TLR-2 signalling kinetics were measured for 2 h in an ELISA reader (Biotek, Bad Friedrichshall, Germany) at OD_640_.

### Statistics

One-way ANOVA multiple comparison test corrected using the Tukey test was performed for unpaired comparisons between the data of WT, Δ*cas9* gene deletion mutants and Δ*cas9*::cas9-complemented mutants. To compare data between GB11 and its GB11Δ*cas9* mutant only, paired T-tests were performed where appropriate. All analyses were performed using Prism software (GraphPad Software Inc., USA), and outcomes with *P*<0.05 were considered statistically significant.

## Results

### CjeCas9 modulates cell envelope integrity of *C*. *jejuni*

**Fig. 1.**
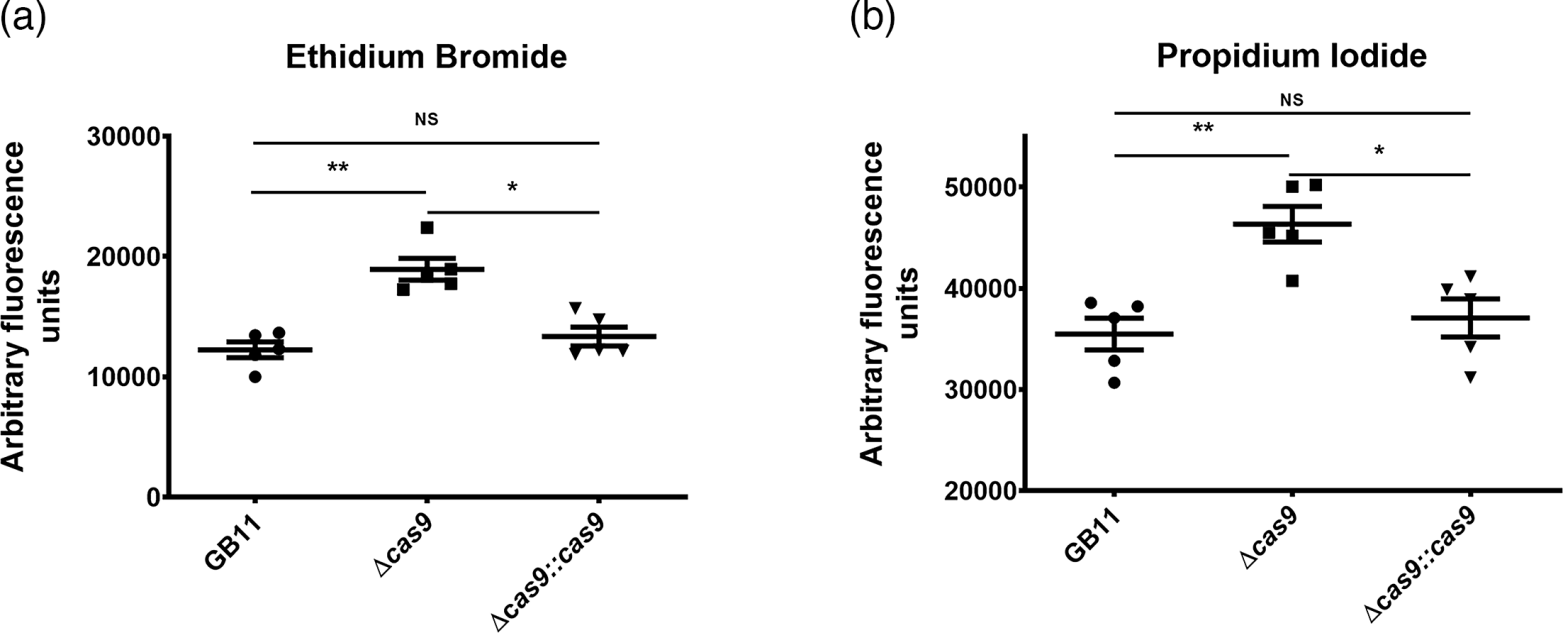
Gene deletion mutant exhibits increased permeability to propidium iodide and ethidium bromide. Exponentially growing GB11 WT, its isogenic ∆*cas9* gene deletion mutant and the ∆*cas9*::cas9-complemented mutant were exposed to vancomycin on BA media (*n*=4), washed and stained with ethidium bromide (**a**) (left panel) or propidium iodide (**b**) (right panel). Fluorescence was measured at 605 nm for ethidium bromide and at 617 nm for propidium iodide. *P*<0.05 was considered statistically significant. **, *P*<0.01; ***, *P*<0.001; ns, not significant. Data are shown as mean±sem. *N*=3–6 samples per scattergram in each graph. Circles represent for GB11 the WT; squares the GB11Δ*cas9* mutant; and triangles the GB11Δ*cas9*::cas9-complemented mutant strain.

In *F. novicida* bacteria, the presence or absence of FnoCas9 affects cell envelope permeability [5]. We therefore investigated whether the cell envelope permeability of *C. jejuni* GB11 was affected by the presence or absence of CjeCas9. To assay membrane permeability, we made use of ethidium bromide and propidium iodide that, once having passed the cell membrane, binds bacterial DNA and emits fluorescence [5]; higher fluorescence intensities correspond with more permeable cell membranes. Staining with ethidium bromide showed that uptake of this compound by the Δ*cas9* gene deletion mutant strain of GB11 was significantly higher (*P*=0.0002) compared to GB11 WT (Fig. 1a). Bacterial cell envelope integrity was further assessed by uptake of propidium iodide, which has a similar ring structure as ethidium bromide but features a linear side chain. Propidium iodide uptake by the GB11Δ*cas9* gene deletion mutant strain had significantly increased (*P*=0.0023) compared to uptake by GB11 WT (Fig. 1b). Complementing the *cas9* deletion by introducing the WT gene in the GB11Δ*cas9* mutant and comparing fluorescence between WT and complemented strain (both producing CjeCas9) showed a nonsignificant difference, indicating restored cell membrane resilience to ethidium bromide (*P*=0.60) and propidium iodide (*P*=0.80) (Fig. 1a, b).

To provide further support for the notion that CjeCas9 apparently influences *C. jejuni* membrane permeability, we performed permeability assays using *C. jejuni* isolate NCTC11168. Staining with ethidium bromide showed that uptake of this compound by the Δ*cas9* gene deletion mutant strain of NCTC11168 was significantly increased (*P*=0.0213) compared to the NCTC11168 WT (Fig. S1A, available in the online Supplementary Material). Although uptake of propidium iodide was elevated in the Δ*cas9* gene deletion mutant compared to NCTC11168 WT, results were not significantly different (Fig. S1B). Complementing *cas9* deletion by introducing the corresponding WT gene in the NCTC11168Δ*cas9* mutant restored cell envelope resilience to ethidium bromide uptake by the complemented mutant to WT levels ((*P*=0.0150) Fig. S1A).

### CjeCas9 affects ciprofloxacin susceptibility

The antibiotic ciprofloxacin, frequently prescribed to treat *C. jejuni* infections [21], has to cross the bacterial cell envelope and accumulate intracellularly to establish its bactericidal effect [29]. Because of the effect of CjeCas9 on the permeability of the *C. jejuni* cell envelope that we reported above, we wondered whether CjeCas9 could be involved in *C. jejuni* tolerance to those classes of antibiotics that have to cross cell membranes before establishing bactericidal effects. We explored the effect of the presence/absence of CjeCas9 on the MIC of ciprofloxacin. *C. jejuni* GB11Δ*cas9* isogenic mutant showed an MIC reduction by a factor of 2 compared to its WT parent strain (*P*=0.0173), suggesting an increased susceptibility to ciprofloxacin (Fig. S2). Complementation of the corresponding WT *cas9* gene into GB11Δ*cas9* restored MIC values to WT values (Fig. S2). This finding for ciprofloxacin was reproduced in the *C. jejuni* isolate NCTC11168 WT and its Δ*cas9* gene deletion mutant and complemented mutant variants (Fig. S2). Compared to its WT parent strain, NCTC11168 Δ*cas9* gene deletion mutant revealed an increased susceptibility of at least a factor of 2 (*P*=0.001).

This differential antibiotic sensitivity depending on production (or not) of CjeCas9 could be partially reproduced for both GB11 and NCTC11168 and their mutant strains to a few other antibiotics including erythromycin and clarithromycin (Fig. S3A–D). For NCTC11168, increased sensitivity of the Δcas9 gene deletion mutant could be complemented for both erythromycin and clarithromycin, but not for the GB11Δ*cas9*::cas9-complemented mutant due to its lab-engineered (to select for complemented mutants) resistance to erythromycin and clarithromycin. For GB11, we tested additional antibiotics, tetracycline, polymyxin B and colistin, showing differential sensitivity of GB11 WT and its Δcas9 gene deletion mutant; these results could not be complemented (Fig. S3E–G).

### Growth assays to investigate the effect of *cas9* on *C. jejuni* strain viability

The measured differences in cell envelope integrity and antibiotic resistance could theoretically result from differential viability of WT and ∆*cas9* deletion mutants. We therefore assayed growth performance of WT and isogenic ∆*cas9* gene deletion mutant strains over time periods of at least 48 h in LB broth (see Experimental procedures). No differences in growth performance were observed for reference isolate NCTC11168 and its ∆*cas9* gene deletion mutant (Fig. S4A). For GB11 WT and its ∆*cas9* gene deletion mutant, a slight difference was observed over time in growth performance that was near-significant after 50 h of growth (*P*=0.0687) (Fig. S4B). These results are in agreement with our earlier results when culturing WT and corresponding ∆*cas9* gene deletion mutants displaying no significant differences in growth performance [24].

### Presence or absence of CjeCas9 affects *C*. *jejuni* swarming

**Fig. 2.**
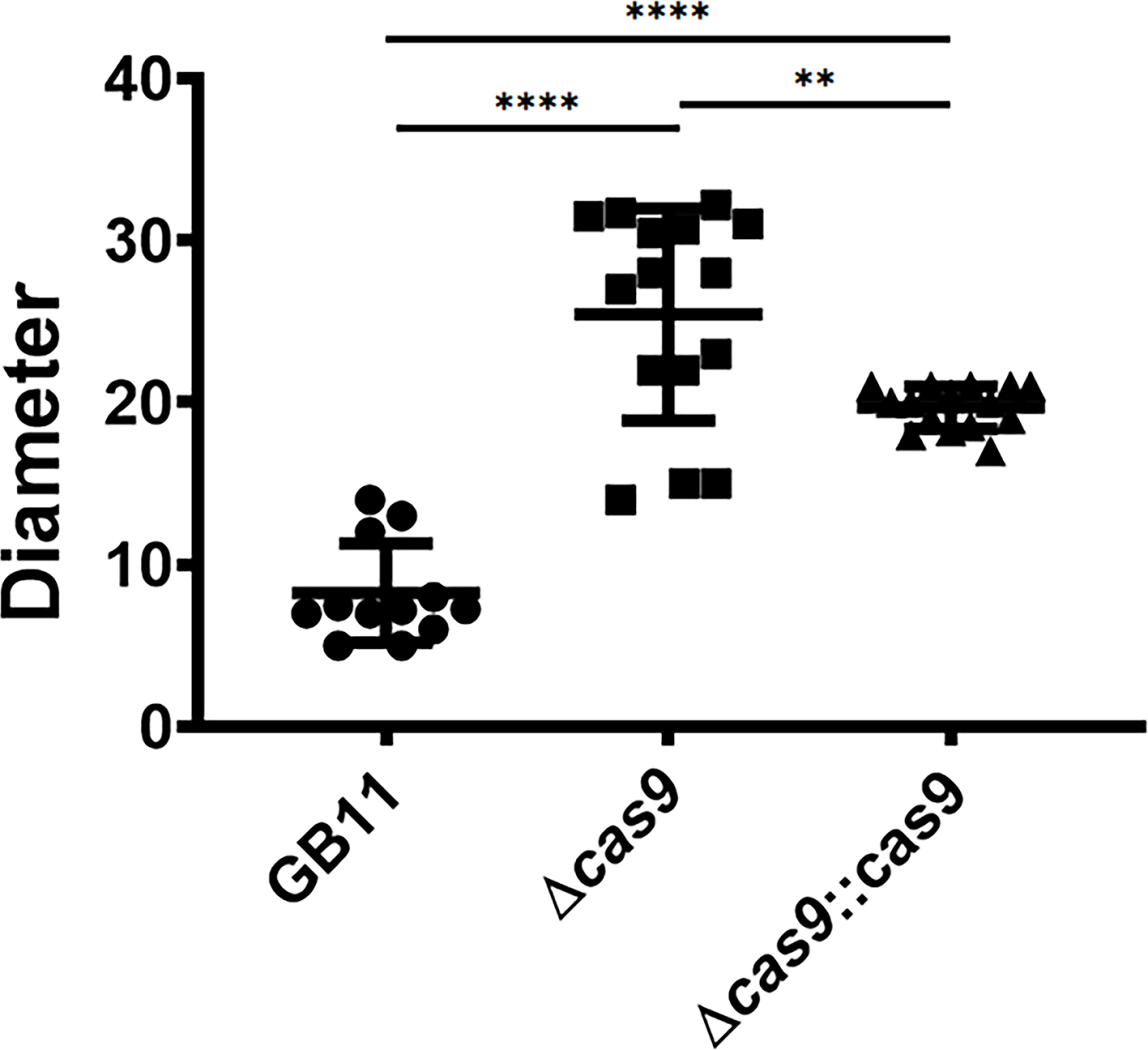
*cas9* gene deletion mutant exhibits increased swarming. GB11 WT, its isogenic ∆*cas9* deletion mutant and the ∆*cas9*::cas9-complemented mutant were stabbed into semi-solid MH agar media. After overnight growth, swarming diameters were measured in millimetres and are displayed here as mean values±sem. *P*<0.05 was considered statistically significant. **, *P*<0.01; ****, *P*<0.0001. Data are shown as mean+sem. *N*=10–15 samples per scattergram in each graph. Circles represent the GB11 WT strain; squares the GB11Δ*cas9* gene deletion mutant; and triangles the GB11Δ*cas9*::cas9-complemented mutant strain.

*C. jejuni* cellular motility, or swarming (in films of liquid on solid media), allows bacteria to move towards nutrients and host factors and is a relevant virulence factor [30]. Increased swarming times are associated with less efficient chemotaxis (coordinated movement in the direction of a chemical factor, for example, a nutrient) which is relevant for efficient host colonization [30]. We previously discovered that loss of CjeCas9 influenced swarming phenotypes of various *C. jejuni* GBS isolates, whereas supplementation of CjeCas9 into Cas9-non-producing strains reduced the increased-swarming time phenotype [24]. We performed swarming assays with strain GB11, its Δ*cas9* gene deletion mutant and GB11Δ*cas9*::cas9-complemented mutant strain as described in Methods and found that deletion of *cas9* in strain GB11 significantly influenced swarming behaviour when compared to WT GB11 strain (swarming diameters: 8.242±2.38 for GB11 WT, 25.44±5.55 for GB11Δ*cas9* gene deletion mutant strain and 19.69±1.11 for the GB11Δ*cas9*::cas9-complemented mutant strain (Fig. 2) (*P*<0.0001)). Complementation of *cas9* in GB11Δ*cas9* isogenic mutant restored swarming diameter phenotype towards WT levels (*P*<0.0019) but swarming diameters of WT and GB11Δ*cas9*::cas9-complemented mutant strains were significantly different (*P*<0.0001) (Fig. 2). Movie clips of GB11 WT and its Δ*cas9* gene deletion mutant reveal that WT and mutant are viable and motile in cell culture medium and that motility of the Δ*cas9* gene deletion mutant appears to be less coordinated (Movies S1 and S2). No visible differences were observed between the movement of the WT NCTC1168 strain and its Δ*cas9* mutant (Movies S3 and S4).

### Loss of production of CjeCas9 increases CT binding to sialylated LOS structures

**Fig. 3.**
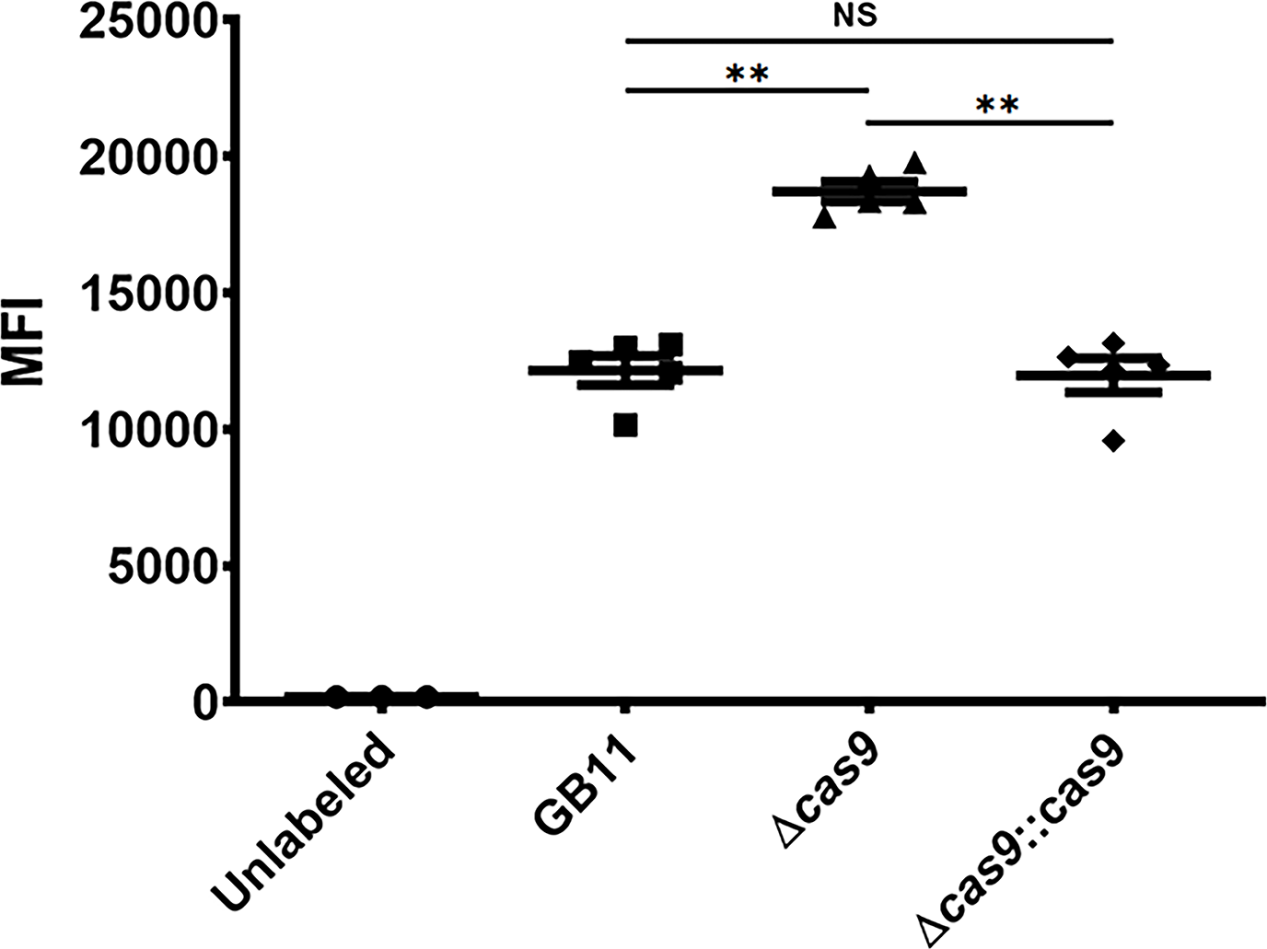
CjeCas9 modulates the production of sialylated LOS structures. Flow cytometric MFI analysis of GB11, its isogenic Δ*cas9* deletion mutant and Δ*cas9*::cas9-complemented mutant strain. The GB11Δ*cas9* mutant showed increased sialylated LOS content of GM1 on the cell envelope, compared to GB11 and the GB11Δ*cas9*::cas9-complemented mutant strain, in CT binding assays. Pooled data of three independent experiments are shown; *P*<0.05 was considered statistically significant. **, *P*<0.01; ns, not significant. Data are shown as mean±sem. *N*=3–6 samples per scattergram in each graph. Circles represent unlabelled *C. jejuni*; squares represent the WT; triangles the Δ*cas9* gene deletion mutant; and diamonds the Δ*cas9*::cas9-complemented mutant strain.

We have shown above that GB11Δ*cas9* showed an increased (not significant) susceptibility to ciprofloxacin (Fig. S2). *C. jejuni* susceptibility to ciprofloxacin is associated with the presence/absence of sialylated LOS structures [21]. GB11 expresses sialylated LOS structures GM1 and GD1a [27]; the presence of GM1 can be detected in CT binding assays [31]. After heat inactivation to expose sialylated LOS structures, the GB11Δ*cas9* strain revealed significant increases in LOS binding by CT compared to GB11 WT (*P*=0.0035) that was restored to WT levels by *cas9* complementation (Fig. 3). This suggests that loss of CjeCas9 production is accompanied by an increased exposure of sialylated LOS structure GM1 on the outer surface of *C. jejuni*.

### CjeCas9 is required for survival inside Caco-2 intestinal epithelial cells

Once we had found that CjeCas9 influenced cell envelope stability, sialylated LOS production and LOS exposure, important virulence traits that mediate bacterial survival inside and transcytosis across epithelial cells [5,7, 17, 25, 28, 32], we decided to investigate if loss of CjeCas9 production negatively influenced host cell infection, transcytosis and/or intracellular survival. To this, we first assessed the invasion potential of GB11, GB11Δ*cas9* and GB11Δ*cas9::cas9* strains. The increase in sialylated LOS as measured in the CT assays (above) made us wonder whether increased invasion and/or survival phenotype of *C. jejuni* was in agreement with our previous findings [28], so we compared the invasion of Cas9-producing and (non-)producing GB11 strains. We found that GB11Δ*cas9* with increased sialylated LOS displayed a reduced invasion potential, compared to its GB11 WT (*P*=0.0084) and complemented GB11Δ*cas9*::cas9 mutant strains (*P*=0.0020), expressed as c.f.u. (Fig. S5).

**Fig. 4.**
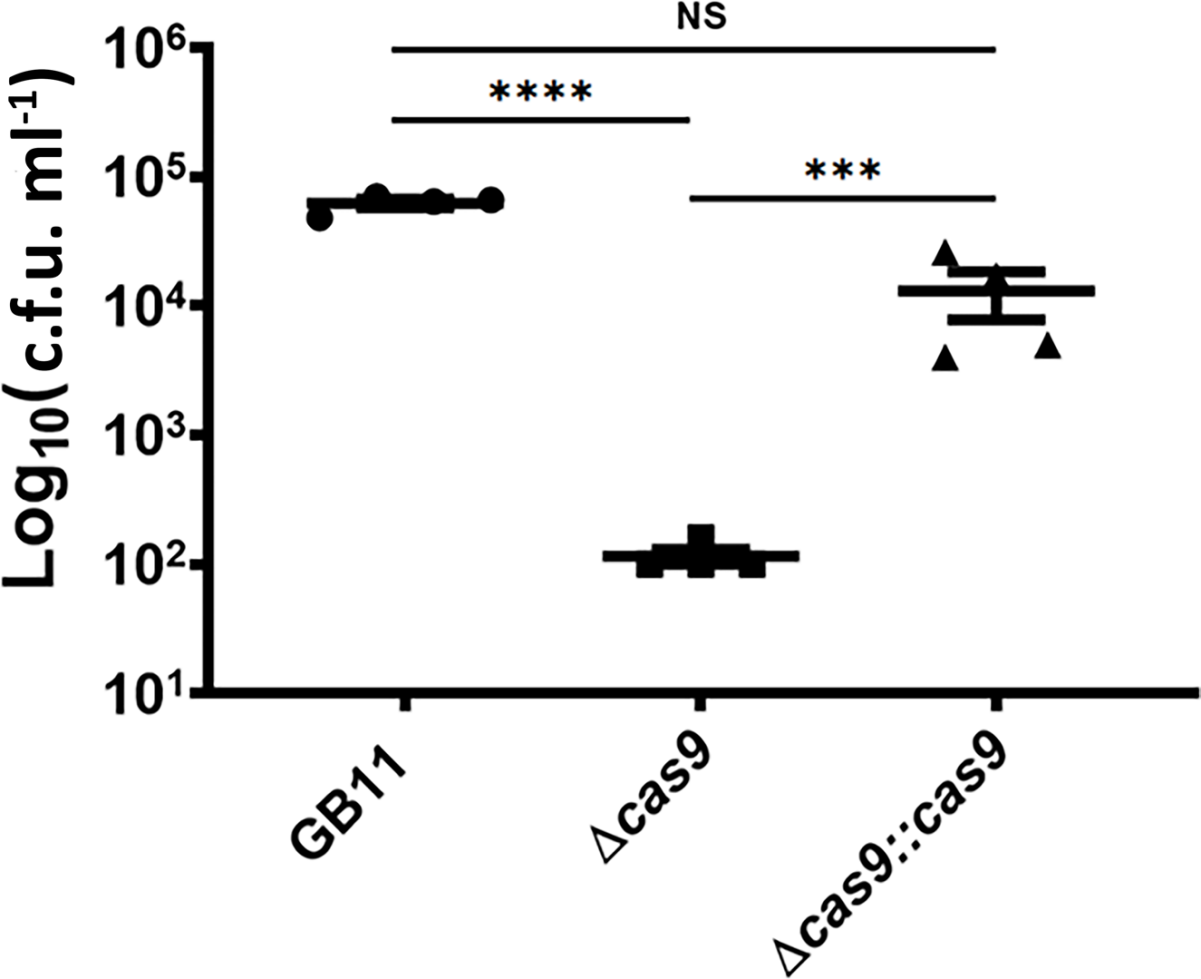
CjeCas9 modulates *C. jejuni* survival in human Caco-2 cells. The potential of *C. jejuni* WT strain GB11, GB11Δ*cas9* deletion mutant and the GB11Δ*cas9*::cas9-complemented mutant to survive inside Caco-2 intestinal epithelial cells. Numbers of c.f.u. were enumerated after 48 h of survival assays. Circles represent the WT GB11; squares the Δ*cas9* mutant; and triangles the Δ*cas9*::cas9-complemented mutant strain. Differences in survival were tested for significance. *P*<0.05 was considered statistically significant. ***, *P*<0.001; ****, *P*<0.0001; ns, not significant. Data expressed as geometric means for at least three independent experiments.

We found that survival in Caco-2 intestinal epithelial cells of GB11Δ*cas9* was a factor of 550 lower compared to survival by the GB11 WT strain (Fig. 4), a significant difference (*P*<0.0001). Complementation of mutant GB11Δ*cas9* confirmed restoration of *C. jejuni* intracellular survival phenotype to near WT levels, suggesting that CjeCas9 is required for intracellular survival of *C. jejuni* and suggesting a potential interplay between Cas9 production, LOS sialylation and *C. jejuni* virulence, as proposed earlier by us [33].

### CjeCas9 modulates endosomal-mediated translocation

**Fig. 5.**
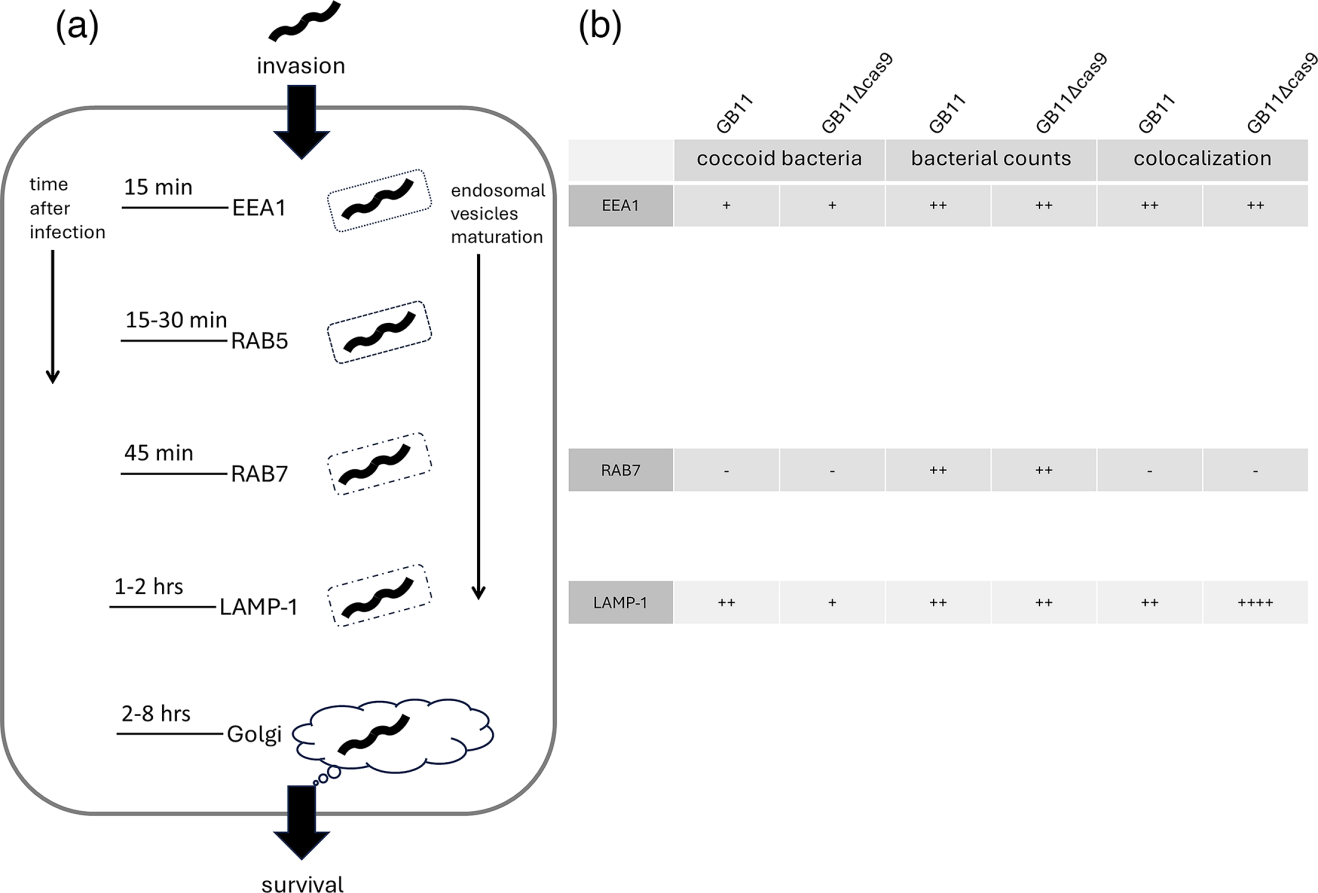
Main steps and time points of invasion of, and escape from, human cells by *C. jejuni* bacteria. (a) *C. jejuni* and the compartment in which this bacterium resides colocalizes with EEA1 after 15–20 min, with RAB5 between 15–30 min. All *C. jejuni* isolates were found to be positive for colocalization with these two endosomal markers. After 45 min during the colocalization with RAB7, the first differences were observed, with some *C. jejuni* isolates colocalizing or not with the endosomal marker RAB7, which affects phagolysosomal maturation and thus intracellular survival. After 2 h, all *C. jejuni* bacteria colocalized with LAMP-1. (b) Data displayed in this figure include next to the presented data and in Figs S3 and S4 data from [16,17].

Earlier, by tracing colocalization of *C. jejuni* with cellular markers associated with endosomal and lysosomal compartments at specific time points [17], we discovered that transcytosis of *C. jejuni* across Caco-2 cells occurred at set periods. Since non-production of Cas9 by the GB11Δ*cas9* gene deletion mutant strain significantly affected intracellular survival in Caco-2 cells, we assayed whether recruitment of the early (EEA1) or late (RAB7 and LAMP-1) endosomal markers correlated with CjeCas9 presence or absence. An outline of the time points and time-associated transcytosis markers for *C. jejuni* invasion of human cells is illustrated in Fig. 5.

In the early phase of cell invasion and transcytosis, within 15–30 min, we found significant differences in numbers of visibly coccoid-shaped (non-invasive) bacteria, the number of *C. jejuni* bacteria per microscopic field and the percentages of EEA1-positive *C. jejuni* containing Caco-2 endosomes, all between WT GB11 and its isogenic GB11Δ*cas9* mutant strain (Fig. S6A–C).

After 45 min, numbers of *C. jejuni* bacteria per microscopic field and coccoid shape had remained significantly different between GB11 and its GB11Δ*cas9* gene deletion mutant strain (Fig. S6D, E). Moreover, deletion of *cas9* in GB11 led to a significantly differential recruitment of late endosomal marker RAB7 to GB11Δ*cas9*-containing Caco-2 vacuoles compared to vacuoles containing WT GB11 (*P*<0.0001) (Fig. S6F). After 2 h, coinciding with the recruitment of the late endosomal/lysosomal marker LAMP-1 to *C. jejuni*-containing endosomes, numbers of *C. jejuni* bacteria per microscopic field and with coccoid shape had remained significantly different between GB11 and its GB11Δ*cas9* gene deletion mutant strain (Fig. S6G, H). Recruitment of LAMP-1 to late endosomal Caco-2 vacuoles containing GB11 or its isogenic GB11Δ*cas9* gene deletion mutant was not significantly different (Fig. S6I).

In summary, CjeCas9 appears to modulate the recruitment of early and late endosomal markers to GB11-positive endosomal Caco-2 vacuoles, via a process that seems to be (largely) independent of the number of bacteria counted per microscopic field. Moreover, (non)production of CjeCas9 is a co-determinant of the shape of GB11 *C. jejuni* bacteria inside Caco-2 cells (Fig. S7). *C. jejuni* bacteria that did produce Cas9 exhibit a typical (for *C. jejuni*) regular helical or spiral shape with flagella wound around their main body. In contrast, *C. jejuni* bacteria that did not produce Cas9 did not exhibit the typical regular spiral shape but rather rod-like or coccoid shapes and appeared degraded in LAMP-1-positive endosomal compartments.

### CjeCas9 modulates TLR-2 activation

Cas9 production by pathogenic *F. novicida* bacteria affected TLR-2-mediated immune recognition of this bacterium [6]. We assessed whether CjeCas9 production affected TLR-2-mediated immune recognition of *C. jejuni*, by measuring TLR-2 activation as absorbance at OD_640_ nm in transgenic human embryonic kidney cells (see Methods). We found that the GB11Δ*cas9* strain activated TLR-2 more than the GB11 WT and complemented GB11Δ*cas9*::cas9 mutant strains (Fig. S8), demonstrating that CjeCas9 is involved in the modulation of TLR-2 activation in human cells.

## Discussion

Earlier, we and others had revealed a crucial role for CjeCas9 in bacterial virulence features and identified a potential mechanism [10,34]. Cas9 proteins produced by *Proteobacteria* influence virulence traits including cell envelope permeability, antibiotic resistance, motility, toxicity towards eukaryotic cells, immune evasion and intracellular survival [5,6, 10, 34,36]. In the present work, we extend these findings by uncovering that CjeCas9 also modulates processes that mediate intracellular survival of virulent *C. jejuni* bacteria inside Caco-2 intestinal epithelial cells. In addition, we discovered the effects of Cas9 production on cell envelope permeability, sialylated GM1 LOS expression, TLR-2 activation, motility (including swarming) and antibiotic resistance against ciprofloxacin, erythromycin and clarithromycin. These features are biologically relevant and clinically important, since they contribute to bacterial fitness and clinical phenotypes associated with *C. jejuni* infections [37]. Differential cell envelope integrity and antibiotic resistance due to differences in cell envelope composition caused by (non-)production of Cas9 are likely correlating with differential viability and virulence. We made sure that non-production of Cas9 did not negatively affect the viability of *C. jejuni*, reproducing previous work by us and others [24,34]. In phage challenge experiments, Δ*cas9* mutants were susceptible to phage infections which required viable bacteria [24]. Others have reported differences in erythromycin sensitivity of strain NCTC11168 and its *cas9* gene deletion mutant [38] similar to our findings we report here. In addition, we revealed increased ciprofloxacin and clarithromycin sensitivity of *cas9* gene deletion mutants of strain NCTC11168; this increased sensitivity could be restored to WT levels by complementation of the *cas9* gene deletion mutant strains.

*Campylobacter* infections range from asymptomatic conditions to severe colitis and bacteraemia [37]. In susceptible patients, these infections are associated with morbidity, mortality and post-infectious complications, with host and bacterial factors being relevant to pathogenesis [14,37]. Bacterial membrane stability, swarming, chemotaxis, motility, sialylated LOS, mucus colonization and susceptibility to antibiotic treatment play important roles in the ability of *C. jejuni* to colonize hosts and affect clinical outcomes in the human host [39]. To reach the intestinal epithelium, *C. jejuni* needs to cross the mucus layer, an event that depends on *C. jejuni* chemotactic responses and motility or swarming [37]. We and others discovered that the absence (non-production) of CjeCas9 affects the motility of *C. jejuni* and the ability to adhere onto, invade in and translocate across intestinal epithelial cells [24,34]. We previously reported that *cas9* gene deletion mutants of GB11 and NCTC11168 showed lower adhesion and invasion compared to their respective WT strains [10]. In contrast, in a Transwell model system in the presence of mucus, differences in adhesion and invasion for *C. jejuni* reference strain NCTC11168 and its *cas9* gene deletion mutant were not observed [24], likely caused by low motility of the used NCTC11168 isolate and the mucus production of the used intestinal epithelial cells. At least two NCTC11168 variants, one motile and one less to non-motile, are circulating in research labs [40], which may explain differences in motility of NCTC111168 strain and its derivatives that we observed in comparison to Shabbir *et al*. [34,38]. Here, we have shown that swarming (motility) diameters of a *cas9* gene deletion mutant of GB11 were larger compared to its isogenic GB11 WT strain, apparently with less coordinated motility. *C. jejuni* motility is driven by its flagella, and their assembly is correlated with membrane biogenesis including LOS production [41], which we show here is also correlated with CjeCas9 production. Of note, LOS type is also influencing the capacity of *C. jejuni* strains to bind to, and translocate across, human intestinal epithelial cells [17]. Additionally, we report here a diminished translocation phenotype of the *cas9* gene deletion mutant of GB11, similar to what we previously found for strain NCTC11168 [10], suggesting that some CjeCas9 activity is associated with *C. jejuni* translocation. We have previously proposed an intertwined interplay between LOS type and sialylation, adhesion and invasion, intracellular survival and CjeCas9 activity [33]. In addition to direct virulence functions of CjeCas9 [10], CjeCas9 may be involved in the regulation of endogenous virulence-associated gene expression [42], as shown for other bacteria [7,25, 32, 43]. In order to unravel the interplay between CjeCas9 production, *C. jejuni* LOS type and sialylation and membrane composition and infection of and translocation across host cells, mechanistic studies to understand how CjeCas9 functions during host–microbe interactions, as have been carried out for *F. novicida* [4,6, 35], are required. For *F. novicida,* it was shown that deletion of *cas9* was associated with an increase in the expression of a lipoprotein [35] and differential regulation of endogenous genes [4]. For *C. jejuni*, in this work and previously [24], we noticed an increased production of sialylated LOS in the absence of CjeCas9. To uncover a Cas9-dependent regulatory mechanism mediating LOS sialylation, it is necessary to assess overall *C. jejuni* regulatory complexity at RNA and protein levels and realize that elucidating unknown regulatory mechanisms will take years, as shown for *F. novicida*.

Additional *C. jejuni* virulence factors, such as secretion of toxins that help to disrupt the cellular function of the intestinal epithelial cells, may play roles during translocation as well [44]. We demonstrated that CjeCas9 is a crucial *C. jejuni* factor that induces programmed cell death (apoptosis) in eukaryotic cells, resulting in the clinical phenotypes associated with *C. jejuni* infections [10,45, 46], and that time to death depends on the respective eukaryotic cell line [45]. Human epithelial cell death phenotypes following CjeCas9-producing *C. jejuni* infections align with patient symptoms, since apoptosis of the intestinal epithelium leads to diarrhoea associated with loss of electrolytes, water and sometimes blood [47,48].

Several *C. jejuni* in host and intracellular survival mechanisms have been unravelled, sometimes together with the corresponding host responses [37,39]. One important intracellular process that contributes substantially to *C. jejuni* survival is interfering with the fusion of *Campylobacter*-containing vacuoles and lysosomes [16]. The entry pathway used by *C. jejuni* bacteria into host cells is one determinant of *C. jejuni* survival, and we have shown previously that LOS sialylation enhanced *C. jejuni* translocation across intestinal epithelial cells [17,28].

In the present work, we revealed that LOS sialylation and intracellular survival of GBS-associated *C. jejuni* strain GB11 are modulated by CjeCas9, in agreement with our previous findings [17,24]. (Non-)production of CjeCas9 affected the expression of sialylated LOS and intracellular survival within Caco-2 intestinal epithelial cells. Earlier, we showed that (non-)production of CjeCas9 by *C. jejuni* strains modulated Caco-2 cellular transcriptomes upon challenge by these strains [45]. Here, we show that non-production of CjeCas9 was associated with a higher incidence of coccoid-shaped *C. jejuni* bacteria. Coccoid shape appears part of the response of *C. jejuni* to (environmental) stressors [49], including the intracellular environment of acidic phagolysosomes [16,50]. We microscopically observed, in fluorescence microscopy images of colocalization of *C. jejuni* GB11 with endosomal markers EEA1, RAB7 and LAMP-1, that Cas9-producing *C. jejuni* strains tolerated intracellular conditions better than Cas9-nonproducing strains [17]. Interestingly, two natural *C. jejuni* isolates, R65 and R104 [24] that encode Cas9 proteins, also displayed rod-like to coccoid morphologies and showed increased colocalization with RAB7 [17], comparable to the GB11 *cas9* gene deletion mutant reported here. We here extended these observations of the contribution of CjeCas9 to bacterial cell shape and intracellular survival by supplementing strain 81-176, a natural pathogenic *C. jejuni* strain without CRISPR-Cas system, with the natural coding sequence of the *cas9* gene contained by strain GB11. The helical shape of *C. jejuni* is positively associated with flagellar function and activity [51]. Indeed, we observed microscopically that non-production of *cas9* is associated with rod-like or coccoid shape and loss of WT flagellar activity and that supplementation of GB11-Cas9 contributed to helical cell shape and flagellar activity. These findings align with previous findings that CjeCas9 appears to be contributing to membrane status, possibly by influencing membrane composition [26] and, as such, directly and/or indirectly contributes to intracellular survival of invading *C. jejuni*.

Different Cas9 regulatory mechanisms linked to virulence have been investigated in various pathogenic bacteria [36], although the exact regulatory mechanism(s) often remained elusive. Cas9 proteins produced by various bacterial pathogens may play direct and indirect (regulatory) roles in virulence traits involved in (invasive) disease processes and survival [35,36]. Indirect regulatory processes at least partially regulated by CjeCas9 might explain our finding that in *C. jejuni*, complementation of *cas9* gene deletion mutants does not always result in restoration of the WT phenotype. For example, for *C. jejuni* GB11, we did not fully restore the WT swarming phenotype when the corresponding *cas9* deletion mutant was complemented, whereas we could reproduce and complement previous findings concerning membrane permeability phenotypes [5]. In all our experiments, *cas9* deletion and complementation had been site-specific, using established procedures [24] and validated by PCR and sequencing. We consider that unknown regulatory mechanisms are associated with the activity of the *C. jejuni* CRISPR-Cas operon and that these mechanisms are disturbed or interfered with during the genetic (DNA) engineering that is part of the genetic complementation laboratory procedures. One candidate mechanism might be, for example, genome-wide DNA methylation patterns, which are associated with expression of virulence and antibiotic resistance in *C. jejuni* strains such as NCTC11168 [52].

Our results reveal direct and/or indirect modulatory role(s) for CjeCas9 in intracellular survival, sialylated LOS expression and antibiotic resistance, biologically relevant features of pathogenic bacteria. Theoretically, CjeCas9 might mediate intracellular survival and antibiotic resistance of *C. jejuni* via endogenous gene regulation, influencing transcription regulators and/or specifically targeting *C. jejuni* genes involved in LOS sialylation. Indeed, during controlled laboratory growth conditions, CjeCas9 binds, and potentially regulates, a wide variety of *C. jejuni* RNAs [42], but to carry out such experiments during infections of eukaryotic cells in controlled, reproducible ways has currently not been possible and would require years of additional experiments at different cellular levels.

In conclusion, we report that CjeCas9 modulates critical *C. jejuni* virulence traits including cell envelope permeability, swarming and bacterial survival inside Caco-2 intestinal epithelial cells. These results show that CjeCas9 not only contributes to virulence by inducing cell death in host cells but also to bacterial survival upon host cell entry and upon exposure to antibiotics.

## Supplementary material

10.1099/mic.0.001638 Uncited Supplementary Material 1.

10.1099/mic.0.001638 video 1.

10.1099/mic.0.001638 video 2.

10.1099/mic.0.001638 video 3.

10.1099/mic.0.001638 video 4.

## Abbreviations

BA: blood agar
Cas9: CRISPR-associated protein 9
CjeCas9: *Campylobacter jejuni* Cas9
CRISPR-Cas: clustered regularly interspaced short palindromic repeats and CRISPR-associated genes
crRNA: CRISPR RNA
CT: cholera toxin
DMEM: Dulbecco’s Modified Eagle’s Medium
HBSS: Hank’s Balanced Salt Solution
hpi: hours post-infection
LB: lysogeny broth
LOS: lipooligosaccharide
MFI: mean fluorescence intensity
MH: Mueller–Hinton
NEAA: non-essential amino acid
TLR-2: Toll-like receptor 2
