# Supplementary material for: Cas9 modulates Campylobacter jejuni virulence traits inside intestinal epithelial cells
Source: Microbiology (Reading). 2025 Dec 5;171(12):001638. doi: 10.1099/mic.0.001638 (PMC12690155; doi:10.1099/mic.0.001638)
Supplement: Uncited Supplementary Material 1. [file mic-171-01638-s001.pdf]

Supplemental figures - Cas9 modulates Campylobacter jejuni virulence traits inside intestinal epithelial cells

Authors: Chinmoy Saha, Dior Beerens, Peter van Baarlen, Rogier Louwen

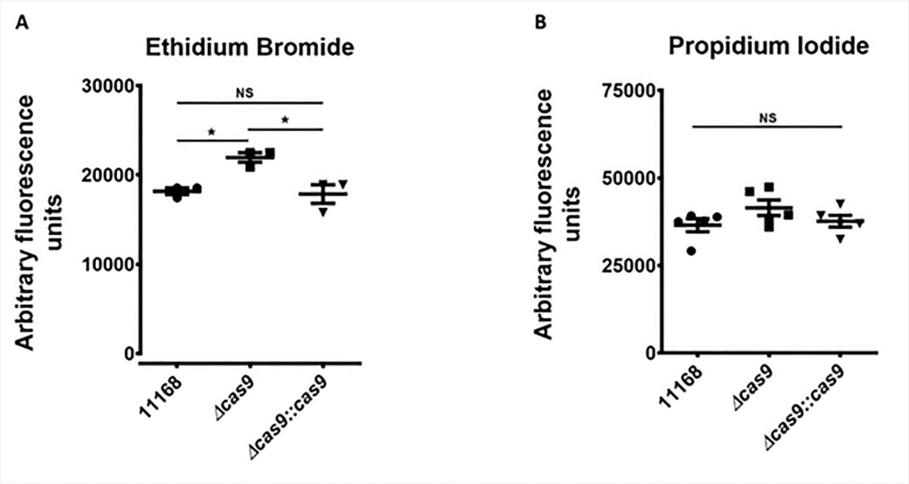

Supplemental Figure 1. NCTC11168 Δ*cas9* gene deletion mutant exhibits increased permeability to ethidium bromide. Exponentially growing NCTC11168 wild type, its isogenic Δ*cas9* deletion mutant and corresponding Δ*cas9*::cas9 complemented mutant were exposed to vancomycin on blood agar media (n=4), washed and stained with ethidium bromide (**A**) (left panel) or propidium iodide (**B**) (right panel). Fluorescence was measured at 605 nm for ethidium bromide and at 617 nm for propidium iodide. $p < 0.05$ was considered statistically significant. * = $p < 0.05$; NS = not significant. Data are shown as mean + SEM. N = 3-6 samples per scattergram in each graph. Circles represent for NCTC11168 wild type; squares the NCTC11168Δ*cas9* gene deletion mutant; and triangles the NCTC11168Δ*cas9*::cas9 complemented mutant strain.

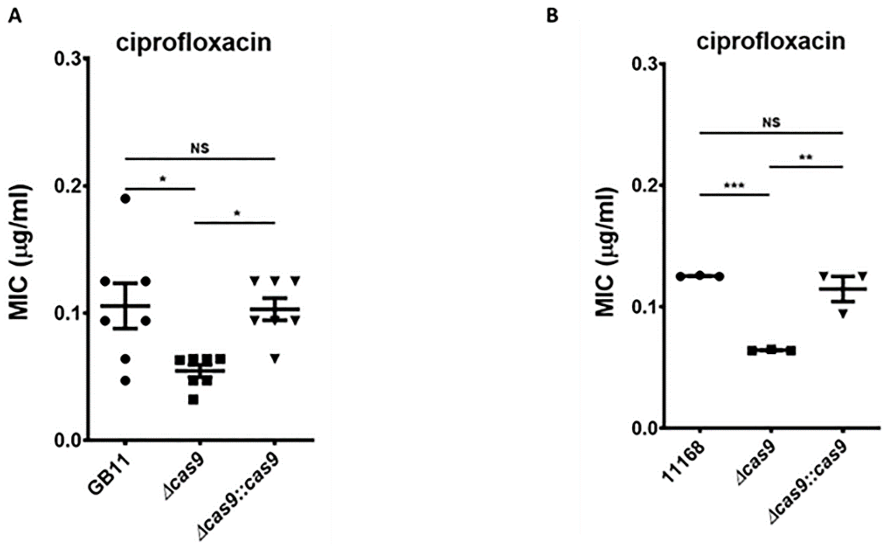

Supplemental Figure 2. *cas9* gene deletion mutant exhibits reduced tolerance to ciprofloxacin. Displayed are the minimal inhibitory concentration **(**MIC) breakpoints as determined by ciprofloxacin E-test for GB11and NCTC11168 wild type, their isogenic Δ*cas9* gene deletion mutants and the corresponding Δ*cas9*::cas9 complemented mutants. Data are shown as mean +/- SEM. N = 3-6 samples per scattergram in each graph. Circles represent the wild type; squares the Δ*cas9* mutant; and triangles the Δ*cas9*::cas9 complemented mutant strain. $p < 0.05$ was considered statistically significant. * = $p < 0.05$, ** = $p < 0.01$, *** = $p < 0.001$, NS = not significant.

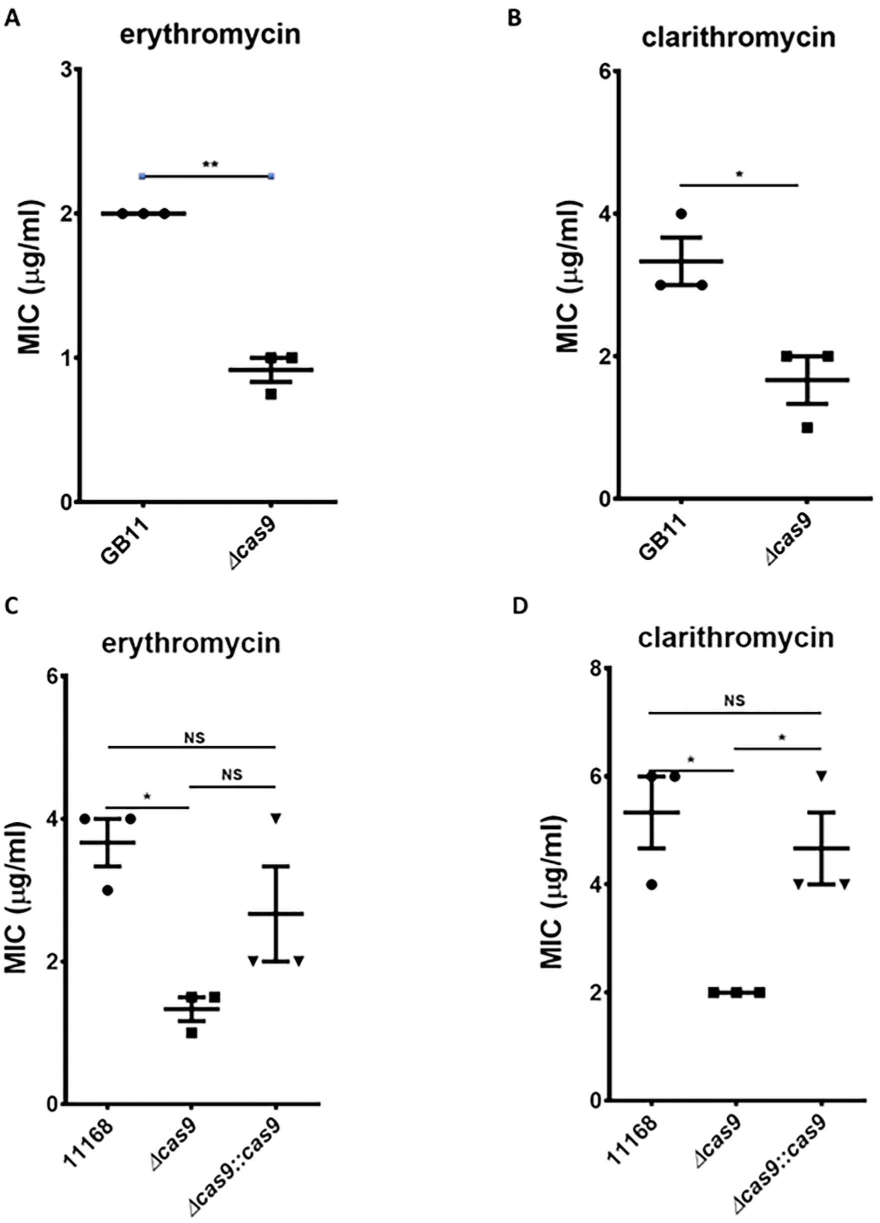

Supplemental Figure 3. Δ*cas9* gene deletion mutants exhibit increased sensitivity to erythromycin and clarithromycin antibiotics. Displayed are the minimal inhibitory concentration (MIC) breakpoints as determined by **A**) erythromycin and **B**) clarithromycin E-test for GB11 wild type and its Δ*cas9* gene deletion mutant. At **C**) and **D**) the resistance for erythromycin and clarithromycin is determined for NCTC11168 wild type, its isogenic Δ*cas9* gene deletion mutant, and the corresponding NCTC11168Δ*cas9*::cas9 complemented mutant. Data are shown as mean +/- SEM. N = 3-6 samples per scattergram in each graph. Circles represent the wild type; squares the Δ*cas9* mutant; and triangles the Δ*cas9*::cas9 complemented mutant strain. $p < 0.05$ was considered statistically significant. * = $p < 0.05$, ** = $p < 0.01$, NS = not significant.

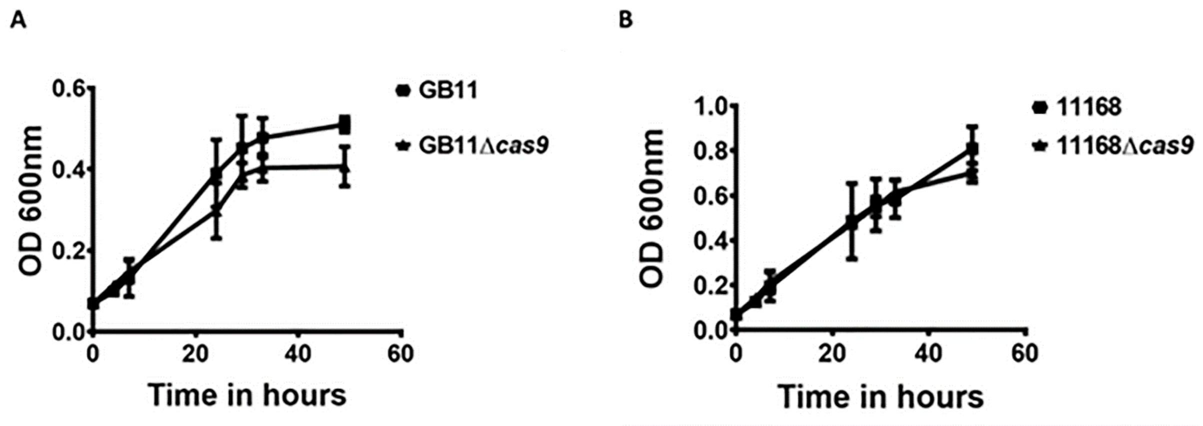

Supplemental Figure 4. Growth Curves of wild-type and corresponding Δ*cas9* gene deletion mutants. Growth curves were determined for **A**) GB11 and its Δ*cas9* gene deletion mutant and **B**) NCTC11168 and its Δ*cas9* gene deletion mutant. Growth was measured at $OD600_{nm}$ up to 50 hours. Two independent measurements were taken per time point during each of 2 independent assays. No significant differences were measured during any of the experiments for either combination of wild-type and Δ*cas9* gene deletion mutant.

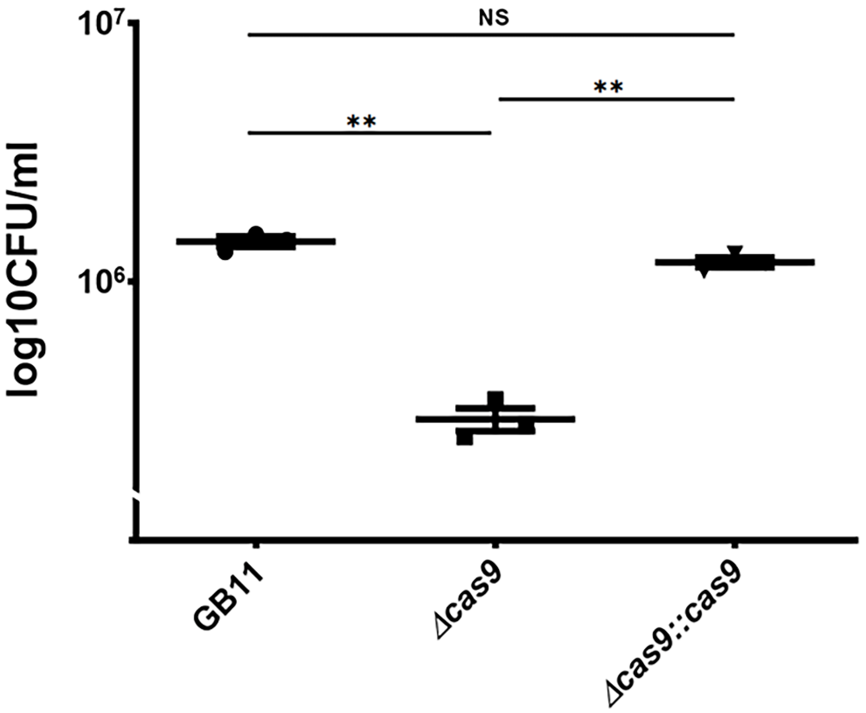

Supplemental Figure 5. CjeCas9 modulates *C. jejuni* invasion into human Caco-2 cells. The potential of *C. jejuni* wild-type strain GB11, its isogenic Δ*cas9* deletion mutant and corresponding Δ*cas9*::cas9 complemented mutant strain to invade intestinal epithelial cells were compared by enumerating numbers of colony-forming units (CFU) on blood agar media after 4 hours of co-incubation of Caco-2 cells and bacteria plus 2 hours gentamicin treatment. Circles represent the wild type; squares the Δ*cas9* deletion mutant; and triangles the Δ*cas9*::cas9 complemented mutant strain. Pooled data of three independent experiments are shown; $p < 0.05$ was considered statistically significant. ** = $p < 0.01$, NS = not significant.

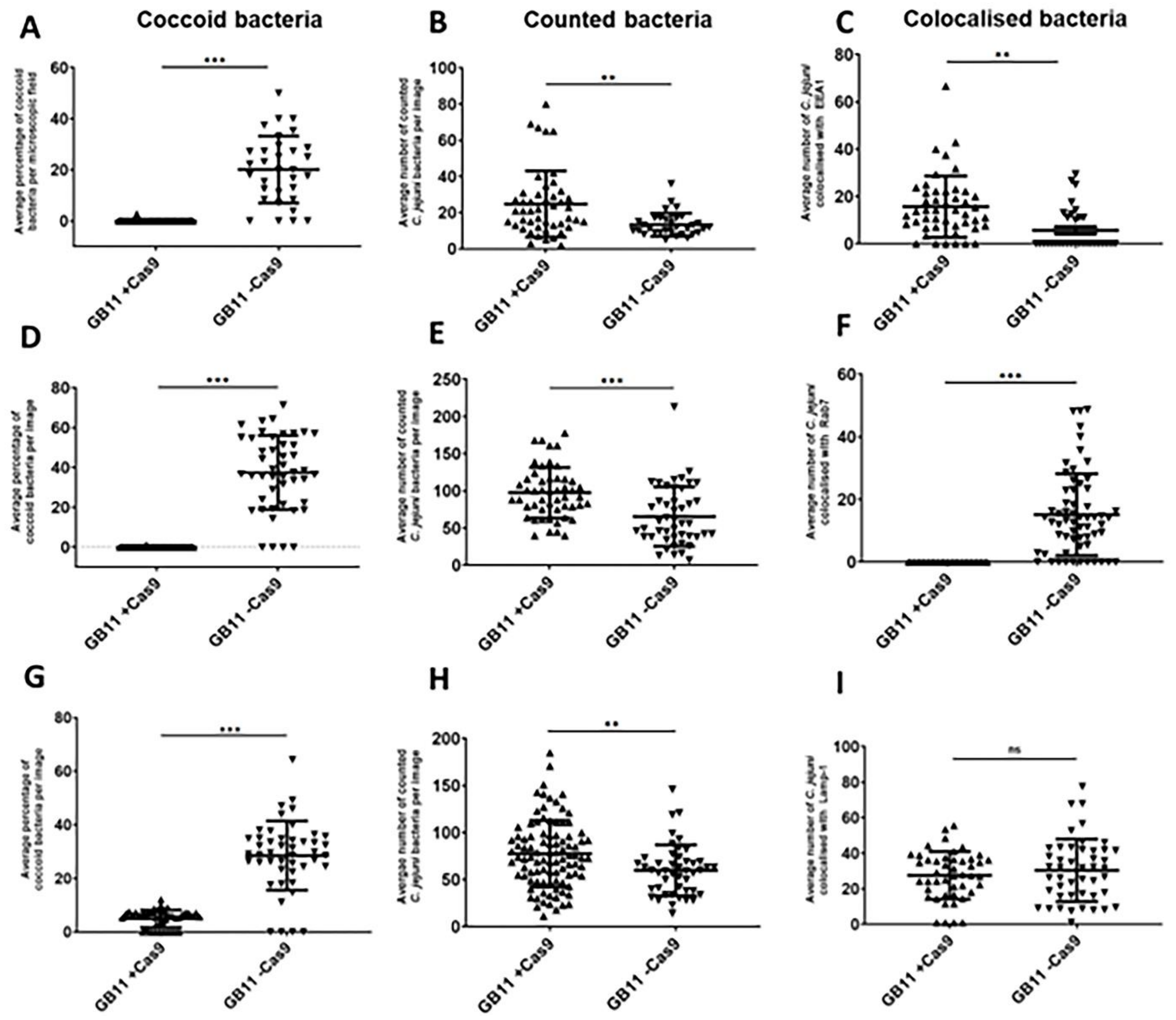

Supplemental figure 6. CjeCas9 modulates the recruitment of endosomal markers

A to C) GB11 *C. jejuni* bacteria that do not produce CjeCas9 colocalize in reduced numbers with EEA1, are significantly more often coccoid shaped, and reveal on average a significant reduced number of bacteria that associate with Caco-2 cells per microscope field. **D to F**) After 45 minutes, the same findings were found for GB11 *C. jejuni* bacteria not producing CjeCas9. CjeCas9-non-producing bacteria associated on average significantly less often with Caco-2 cells per microscope field. CjeCas9-non-producing bacteria appeared more often coccoid shaped and demonstrated a significantly increased co-localisation with RAB7. **G to I**) After two hours the same findings were found for GB11 *C. jejuni cas9* gene deletion mutants. GB11Δ*cas9* associated on average significantly less often with Caco-2 cells per microscope field and presented themselves significantly more often as coccoid-shaped. GB11Δ*cas9* bacteria did not significantly differentially co-localise with LAMP-1 phago-lysosomal markers. $p < 0.05$ was considered statistically significant using a One-Way ANOVA test. ** = $p < 0.01$, *** = $p < 0.001$, NS = not significant. N = a minimum of 30 pictures analysed per scattergram in each graph. GB11 triangles pointing up refer to WT and GB11 and triangles pointing downwards refer to the GB11Δ*cas9* mutant.

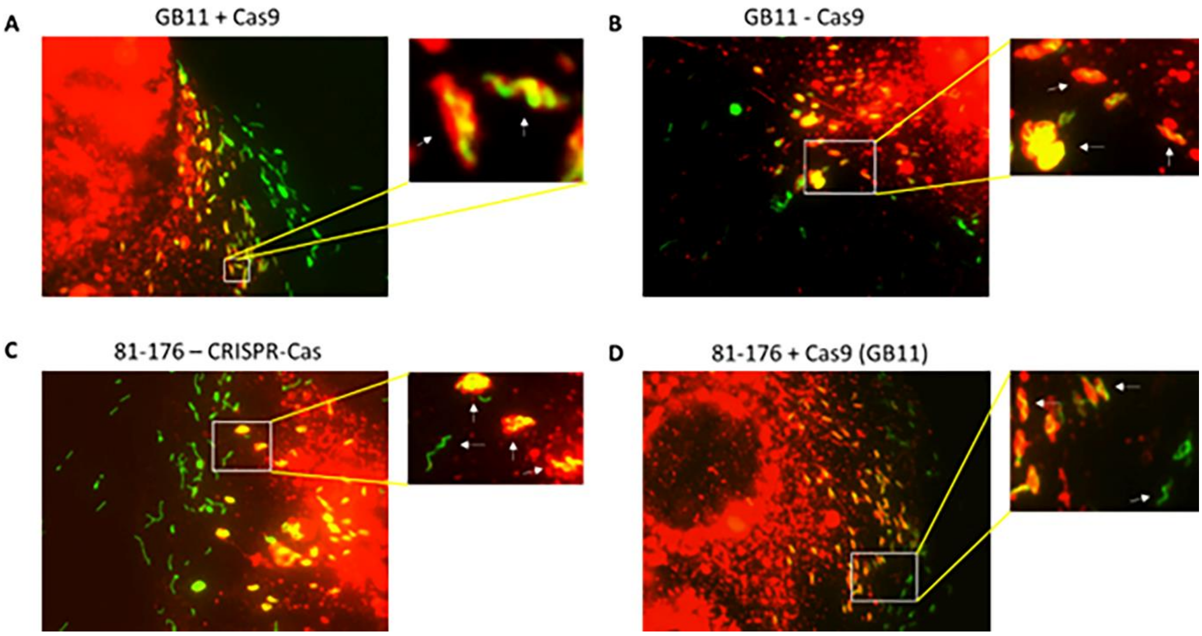

Supplemental Figure 7. CjeCas9 affects bacterial shape inside LAMP-1 positive endosomal compartments. Visualization of the co-localisation of *C. jejuni* bacteria in green that do produce, or do not produce, CjeCas9 with the endosomal marker LAMP-1 in red, in Caco-2 cells, after 120 min of inoculation. The pictures display Caco-2 intestinal epithelial cells, infected by wild-type GB11. Images show spiral-shaped *C. jejuni* bacteria with flagella wrapped around the main body (normal phenotype) inside LAMP-1 positive endosomal compartments, or show bacteria attached to (outside) Caco-2 cells. LAMP-1-detecting antibodies fluoresce red, and *C. jejuni* bacteria-detecting antibodies fluoresce green. When *C. jejuni* and LAMP-1 antibodies co-localise, the combined red and green fluorescent signals turn yellow. Pictures represent Caco-2 intestinal epithelial infections by **A**) GB11 wild type and **D**) 81-176 supplemented with the endogenous GB11*cas9* gene (both producing Cas9) that display typical cellular *Campylobacter* morphology inside LAMP-1 positive endosomal compartments (white arrows). **B**) wild-type 81-176 and **C**) GB11Δ*cas9* bacteria that both do not produce Cas9 display rod-like or coccoid cellular morphology and frequently appear degraded inside LAMP-1 positive endosomal compartments (white arrows).

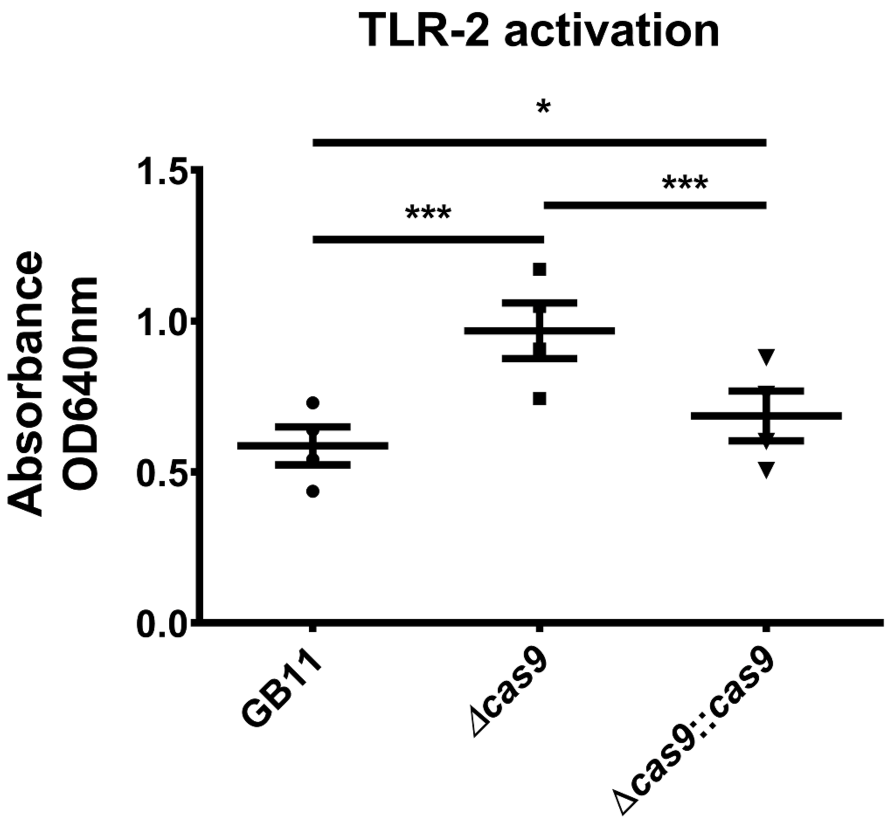

Supplemental Figure 8. CjeCas9 modulates TLR-2 activation by *C. jejuni*. TLR-2 activation by *C. jejuni* wild-type strain GB11, its Δ*cas9* gene deletion mutant strain, and the Δ*cas9*::cas9 complemented mutant strain was measured for 2 hours. Data are shown as mean +/ SEM. $p < 0.05$ was considered statistically significant. * = $p < 0.05$, *** = $p < 0.001$. N = 9 measurements per strain. Circles represent wild type GB11; squares the corresponding Δ*cas9* mutant strain; and triangles the Δ*cas9*::cas9 complemented mutant strain.
